# Interobserver reproducibility of radiographic evaluation of lumbar spine instability

**DOI:** 10.1590/S1679-45082016AO3489

**Published:** 2016

**Authors:** Saulo de Tarso de Sá Pereira Segundo, Edgar Santiago Valesin, Mario Lenza, Durval do Carmo Barros Santos, Laercio Alberto Rosemberg, Mario Ferretti

**Affiliations:** 1 Hospital Israelita Albert Einstein, São Paulo, SP, Brazil.

**Keywords:** Low back pain, Spondylolisthesis, Joint instability/radiography, Lumbosacral region/radiography

## Abstract

**Objective:**

To measure the interobserver reproducibility of the radiographic evaluation of lumbar spine instability.

**Methods:**

Measurements of the dynamic radiographs of the lumbar spine in lateral view were performed, evaluating the anterior translation and the angulation among the vertebral bodies. The tests were evaluated at workstations of the organization, through the Carestream Health Vue RIS (PACS), version 11.0.12.14 Inc. 2009^©^ system.

**Results:**

Agreement in detecting cases of radiographic instability among the observers varied from 88.1 to 94.4%, and the agreement coefficients AC1 were all above 0.8, indicating excellent agreement.

**Conclusion:**

The interobserver analysis performed among orthopedic surgeons with different levels of training in dynamic radiographs of the spine obtained high reproducibility and agreement. However, some factors, such as the manual method of measurement and the presence of vertebral osteophytes, might have generated a few less accurate results in this comparative evaluation of measurements.

## INTRODUCTION

Lumbar pain is an important complaint in daily medical practice, and is present in about 58% of individuals at some time during their lives, and in 36% of people in a one-year period. Most of the time, it presents as acute and self-limited crises.^(1)^ Chronic lumbar pain, accompanied or not by irradiation to the lower limb, which characterizes sciatic pain, has different etiologies, including degenerative instability of the spine, which is highly prevalent.^(2–4)^ Spinal instability is defined by loss of the spine capacity to maintain its shift patterns in physiological conditions, which may cause pain and functional incapacity.^(2)^

Dynamic radiographs of the spine in flexion-extension are simple, low cost, and non-invasive methods of diagnosing instabilities. The presence of abnormal movement in sagittal translation or segment angulation in this evaluation means radiographic instability of the spine.^(4,5)^ Different methods of evaluation are suggested for radiographic assessments. Manual, digitized, and other practically automatic instruments, with the help of computerized overlapping models, are some alternatives. Many authors attribute several values to characterize translational and angular instability. Some studies also observe variations in height of the anterior and posterior disc space, in flexion and extension positions for this analysis, although there is no consensus in the literature reviews about this evaluation.^(4,6,7)^

The correlation between cause and consequence between spinal instability and symptoms is controversial. Various authors associated radiographic spinal instability to severity of symptoms and the need for intervertebral fusion or arthrodesis for definitive treatment.^(4,8,9)^ The isolated presence of radiographic instability is not necessarily related to manifestation of clinical signs and symptoms, and vice-versa. To make a decision about the best management to be individually applied, the specific complaint of the patient, together with the radiological findings, should be considered.^(2,10)^

## OBJECTIVE

To measure the interobserver reproducibility of radiographic evaluation of lumbar spine instability.

## METHODS

This is a study developed at the *Hospital Israelita Albert Einstein* and approved by the Research Ethics Committee of the organization, under protocol CAAE: 47375315.3.0000.0071, Official Opinion number 1.204.567, and is exempt from having an Informed Consent Form. It was designed as an (interobserver) reproducibility measuring study of the radiographic evaluation of lumbar spine instability in vertebral segments L4/L5 and L5/S1. During the period between January 2014 and May 2015, lumbosacral radiographs were randomly selected by an independent observer in the anteroposterior and profile views, in neutral and dynamic positions (extension and flexion). We used the following exclusion criteria: radiographs of patients with acute vertebral trauma, pathological fracture in the lumbosacral region, or any other prior condition by various etiologies, such as neoplasms or infections, which could interfere in the normal anatomy of the region; radiographs of patients with immature skeletons, or with congenital or idiopathic deformities; prior fracture of the spine; poor quality tests; patients submitted to lumbosacral spine operations.

### Radiographic tests

Patients were submitted to lumbar spine radiographs in anteroposterior and side views, in maximal flexion and extension positions, as per the technique originally described by Knutsson^(11)^ to evaluate intersegment instability, later characterized by Putto et al.^(12)^ To perform the flexion radiograph, the patient would sit on a bench or high support, so that the feet were completely supported on the floor, and knees and hips were in slight flexion. The arms remained crossed over the chest. In this way, the patient was instructed to flex the trunk anteriorly as far as possible to get the image at this point. For the image in extension, the patient had to remain in an orthostatic position with arms crossed over the chest, and then the patient was instructed to perform maximal active extension to capture the image at that moment. Measurements were made of the dynamic lumbar spine radiographs in side views, assessing anterior translation and angulation between the vertebral bodies. The tests were evaluated at workstations of the organization, by means of the Carestream Health Vue RIS (PACS), version 11.0.12.14 Inc. 2009^©^ system.

### Description of the measurements

Measurements of sagittal intersegment translation and rotation or intervertebral angulation were made according to the classic methods described in the literature to assess instability.^(13–16)^ Thus we considered instability as of sagittal angulation values >15° between L4/L5 and between L5/S1, as well as values in millimeters of translation on the sagittal plane >4mm or shift >15% of the inferior vertebral endplate measure. The segment was considered unstable when it presented with rotation or sagittal translation greater than the values described. Instability, in the present study, was evaluated in segments L4/L5 and L5/S1, due to greater clinical applicability, since these levels are most frequently affected in degenerative diseases of the lumbosacral spine.^(17–20)^

**Figure 1:**
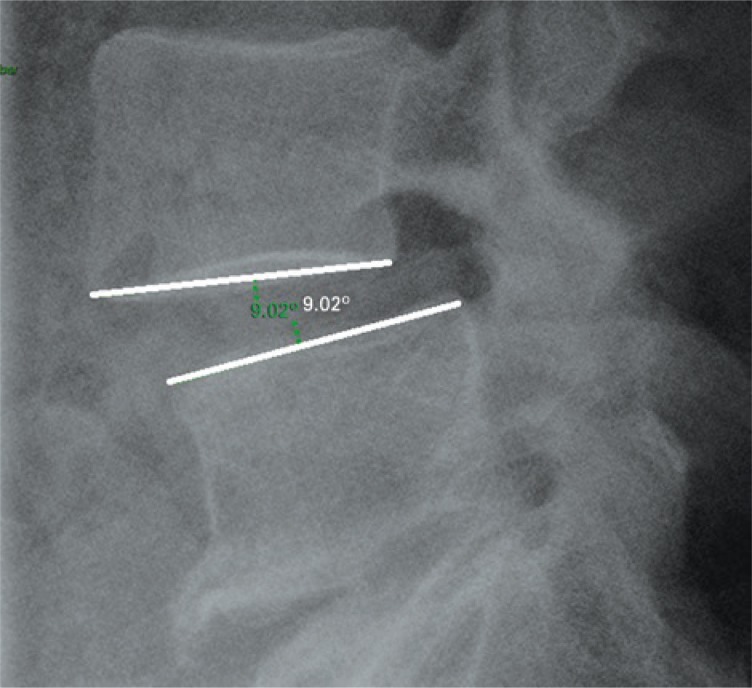
Sagittal rotation angle (L4/L5)

The sagittal rotation angle for each mobile vertebral segment (L4/L5 and L5/S1) was calculated by means of the difference between the Cobb angle values (formed between the line tangent to the inferior endplate of the upper vertebra and the tangent of the superior endplate of the lower vertebra) obtained in dynamic radiographs, in a profile flexion-extension view^(6,21)^ (Figure 1).

**Figure 2:**
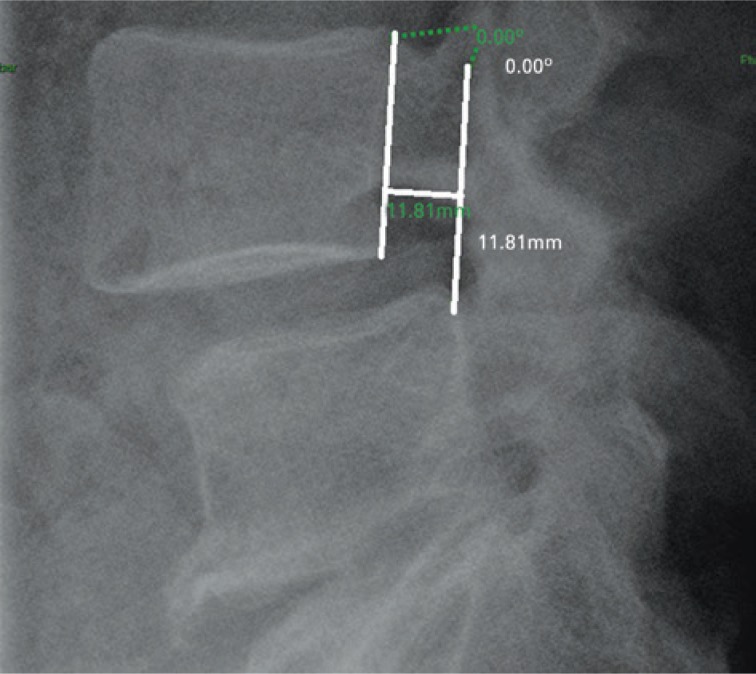
Sagittal translation measurement (L4-L5)

Angulation values were considered positive as this verification determined the lordotic angle; while the negative values were considered as the assessment between the vertebral endplates determined the kyphotic angles. Variation measurements were made by going from extension to flexion. Sagittal translation in each segment was calculated by means of shift measuring, in millimeters, of the superior vertebra relative to the inferior, obtained in flexion-extension radiographs, starting with extension to flexion (Figure 2). For translation, the measures were considered positive when the posteroinferior limit of the superior vertebral body was anterior to the posterosuperior limit of the adjacent inferior vertebra (listhesis). The measures were considered negative when the position of the posterosuperior limit of the superior vertebra was posterior to the posterosuperior limit of the inferior vertebra (retrolisthesis).

We carried out a supplementary evaluation of dynamic intervertebral anteroposterior shift on the sagittal plane, using the measurement of the shift verified, in millimeters, relative to the anteroposterior measurement observed on the corresponding inferior vertebral endplate. The objective was to avoid radiographic magnification bias and obtain greater accuracy, in conformity with the proportionality of the variable size of the vertebrae individually, in each patient.^(7)^ For this analysis, we used the mean value obtained between each radiograph evaluated, in neutral position, flexion, and extension. Values that surpassed 15% of the mean vertebral endplate measurement characterized instability. Despite presenting limitations to the dynamic analysis in flexion-extension, currently the remains as the most common standard reference for the diagnosis of structural lumbar segment instability.^(4,22,23)^

### Observers

Two observers made an analysis of the radiographic tests. Their specialties were orthopedic surgeon specialized in the spine – with six years of practice (observer 1) and a third-year orthopedics resident (observer 2). The images were evaluated and classified by both observers after random selection and exclusion of irrelevant cases by an independent observer who was also an orthopedic surgeon.

### Sample size

To calculate the sample size, we considered as primary objective the estimate of a 95% confidence interval for the Kappa index modified by Fleiss et al.^(24)^ Taking into account an absolute precision of 5%, we estimated that 130 tests should be observed. However, we excluded four tests that did not contain three images necessary for the case to be evaluated in the database, thus totaling 126 patients.

### Statistical analysis

For the interobserver agreement analysis, we used the proportion of agreement, and the coefficients Kappa, by Cohen, and agreement analysis (AC), AC1 by Gwet;^(25,26)^ the p values of and 95% confidence intervals were jointly presented. The agreement coefficients offer and correct the pure matching proportion of agreement between observers. The coefficient value varied from −1 to +1. When the value is −1, the significance is of total disagreement; when the value is +1, it indicates total agreement; and when the value is zero, it represents non-agreement. The values may also be arbitrarily attributed to subdivisions for parametric assessments of agreement, with values of up to 0.20, indicating unsatisfactory agreement; between 0.21 and 0.40, little agreement; between 0.41 and 0.60, moderate agreement; between 0.61 and 0.80, satisfactory and adequate agreement; and values over 0.80 represent almost excellent agreement.^(27,28)^ The software used was R, version 3.0.3, and the significance level adopted was 5%.

## RESULTS

The radiographic tests of 126 patients were observed. For level L4/L5, considering as unstable those with a difference between dynamic vertebral translation values >4mm, observer 1 identified 18 cases of sagittal instability (14.3%) and 14 cases (11.1%) of angular or rotational instability, and the angular differences between extension and flexion >15° for this spinal level. Observer 2 identified 7 cases of sagittal instability (5.6%) and 9 cases (7.1%) of angular or rotational instability. For the analysis of values based on the relation between millimeters observed in translational instability and the mean value of the measurements of the vertebral L5 endplates, determining positivity when results ≥15%, both observers found four cases consistent with instability (3.2%), noted as per this methodology.

For level L5/S1, observer 1 identified 10 cases of sagittal instability (7.9%) and 19 cases (15.1%) of angular or rotational instability. Observer 2 identified 11 cases of sagittal instability (8.7%) and 6 cases (4.8%) of angular or rotational instability. For value analysis based on the relation between millimeters observed in translational instability and the mean value of the measurements of the S1 vertebral endplate, observer 1 found four cases consistent with instability (3.2%), and 3 cases (2.4%) were pointed out by observer 2. The results are shown on tables 1 e 2.

**Table 1:** Description of frequency of instabilities observed

Region	Instability	Observer 1		Observer 2	
		Present n (%)	Absent n (%)	Present n (%)	Absent n (%)
L4-L5	Sagittal	18 (14.3)	108 (85.7)	7 (5.6)	119 (94.4)
	Angular	14 (11.1)	112 (88.9)	9 (7.1)	117 (92.9)
	Based on endplates	4 (3.2)	122 (96.8)	4 (3.2)	122 (96.8)
L5-S1	Sagittal	10 (7.9)	116 (92.1)	11 (8.7)	115 (91.3)
	Angular	19 (15.1)	107 (84.9)	6 (4.8)	120 (95.2)
	Based on endplates	4 (3.2)	122 (96.8)	3 (2.4)	123 (97.6)

**Table 2:** Agreement analysis between observers

Region	Instability	PA	Kappa (95% CI)	p value*	AC1 (95% CI)	p value†
L4-L5	Sagittal	0.90	0.43 (0.28-0.59)	<0.001	0.87 (0.80-0.95)	<0.001
	Angular	0.93	0.57 (0.40-0.74)	<0.001	0.91 (0.86-0.97)	<0.001
	Based on endplates	0.94	-0.03 (-0.21-0.14)	0.713	0.93 (0.88-0.98)	<0.001
L5-S1	Sagittal	0.90	0.32 (0.15-0.50)	<0.001	0.88 (0.81-0.95)	<0.001
	Angular	0.88	0.35 (0.21-0.50)	<0.001	0.86 (0.78-0.93)	<0.001
	Based on endplates	0.94	-0.03 (-0.2-0.14)	0.751	0.94 (0.90-0.99)	<0.001

* p value in reference to the null hypothesis: Kappa =0;

† p value in reference to the null hypothesis.

PA: proportion of agreement;

95%CI: 95% confidence interval;

AC: agreement coefficient.

The agreement in detection of cases of radiographic instability between the observers varied from 88.1 to 94.4%, and the agreement coefficients AC1 were all above 0.8, indicating excellent agreement (Table 1).

## DISCUSSION

Although some authors recommend the limited use of dynamic radiographs of the vertebral spine to determinate instability and possible definition of surgical management in cases of lumbar pain, its application, along with the other supplementary imaging methods, and with a careful physical examination, allow diagnostic and therapeutic help for an adequate treatment of these patients.^(23,29–32)^ For Panjabi, the criteria presented for sagittal instability between L4 and L5, and between L5 and S1 are values >4.5mm; and angular instability >20° in a dynamic evaluation of L4/L5 and >25° between L5/S1.^(2)^ Hence, today there is not an exact concept about the most precise values and technique in this analysis. However, the literature points to results indicating fusion or arthrodesis as a favorable option for treating patients with confirmed lumbar pain and radiographic instability greater than 4mm on the sagittal plane, and 10° on an angular evaluation, according to Hanley criteria for vertebral instability.^(13)^ The accuracy of measurements may also show variations according to the experience and level of training of the observers. Iguchi et al.,^(4)^ in a study directed at evaluating vertebral instability by means of dynamic radiographs of 1,090 patients, used three distinct observers. The mean interobserver difference obtained was <0.5mm for sagittal translation, and <2° for angulation. The agreement index between translation and angulation measurements varied between 0.81 and 0.95. The method was considered reliable with a high degree of reproducibility, since minimal value differences between the measurements do not have significant clinical repercussions.

Among the factors that interfere in a precise radiographic analysis, we point out: the wide age range observed in some studies, the diverse methods of evaluation that compromise the accuracy of measurements, the insufficient sample number, and the inclusion of poor quality radiographs. By means of their results, they also indicate that sagittal translation has a greater influence relative to segment angulation in symptoms; and the concomitance of both radiographic findings point to more exuberant and persistent symptoms. In a study by Pearson et al.,^(23)^ 30 sets of dynamic radiographs were evaluated by three residents in orthopedic surgery; the assessments with digital radiographs, by a computerized manual method, determined, in the interobserver analysis, a total agreement between 79 and 81%, including variations of the anterior and posterior disc height. The values were calculated and compared in this same study by three technical observers who were not physicians. These observers used software that digitized the image and automatically determined specific standards for each vertebra, based on image overlapping techniques. Hence, there was a relative improvement in agreement between 95 and 98%. The authors concluded that small variations in instability measurements, based on manual radiographic assessments, are determined by means of this method, and that technological devices may be incorporated to increase the precision of measurements of vertebral instability. Cakir et al.,^(33)^ in an analysis of angular vertebral instability, based on Cobb method, between L4/L5 and L5/S1, in 24 pairs of radiographs in flexion and extension, verified between two experienced observers a coefficient of correlation of 0.92; whereas the same comparison of results of assessments done by an inexperienced observer and one that was experienced determined a coefficient of correlation of 0.79.

We found no similar studies in the Brazilian literature involving assessments of dynamic lumbar spine radiographs, including a number with statistical relevance (126 patients in the present study) and observer orthopedic surgeons with different levels of experience. Nonetheless, Sperandio et al.,^(34)^ performed assessments of the angle of Cobb in 17 individuals. Measurements were made by an experienced orthopedic surgeon and two physical therapists with no experience in radiographic measurements. The magnitude of the correlation coefficient varied from good to excellent at the thoracic and thoracolumbar levels. In the lumbar spine, there was no significant interobserver correlation, possibly because of a small number of patients with localized curves in this topography. Teixeira et al.,^(35)^ in a study including 40 cases of metastatic vertebral disease and evaluation of stability using the scale Spinal Instability Neoplastic Score (SINS), applied to 17 physicians with different training and levels of experience, found that interobserver agreement was exclusively elevated between experienced spine surgeons. The results were considered poor among other specialists. Pratali et al.,^(36)^ investigated reproducibility between spine surgeons as to definition of treatment of metastatic vertebral lesions, taking into consideration the mechanical stability of the lesions. Twenty cases of isolated metastatic vertebral lesions were presented to 10 specialists, and there was poor interobserver reproducibility in the decision regarding treatment of the metastatic vertebral lesions, when considering the stability of the lesions. Gotfryd et al.^(37)^ assessed vertebral fusion by means of the interobserver analysis of 20 postoperative radiographs of patients submitted to posterolateral arthrodesis of the lumbosacral spine. Six orthopedic surgeons carried out the assessments. Statistical analysis demonstrated weak and poor interobserver agreement for most of the cases. Thus, studies that include observers with different skills and experiences present with varied results in agreement analyses of radiographic assessments, when addressing deformities, neoplasms, or degenerative diseases.^(35–37)^

We consider the present study interesting, since it compares results obtained in assessments performed by two physicians of the same specialty, both orthopedic surgeons, but with different levels of training and experience. The less experienced observer in general detected fewer cases of instability as compared to the observer with training and subspecialization in vertebral spine diseases. Even so, the agreement values reached (0.86 and 0.94) were considered excellent. We determined that for both observers, the cases of sagittal instability based on the measurements of the vertebral endplate were significantly lower relative to the cases determined exclusively by values of translational shift. Possibly, the presence of osteophytes on many radiographs evaluated led to conflicting results in this sense, overestimating the true diameter of the corresponding vertebral endplate, and thus characterizing a likely bias in this method of evaluation.

The possible limiting factors observed in this study are the number of patients, which, in a future analysis, might be increased in order to obtain more precise results, and the wide sample age range, besides observation at a single time point. An intraobserver analysis, at a different time of the primary analysis, could also add new data and collaborate in the individual comparative analysis of the results.

A future study that correlates the degree of radiographic instability with the intensity of symptoms of the patients, may also contribute towards a greater understanding of this complex relationship, besides indicating a possible tendency to greater clinical limitations present in cases of sagittal instability, in comparison with angular instability, as some studies in literature point out.^(4,38)^

## CONCLUSION

The interobserver analysis performed between orthopedic surgeons with different levels of training using dynamic radiographs of the vertebral spine obtained a high level of reproducibility and agreement, even though some factors, such as manual method of assessment and the presence of vertebral osteophytes might have generated a few less consistent results in this comparative evaluation of measurements.
